# Impact of microparticles released during murine systemic inflammation on macrophage activity and reactive nitrogen species regulation

**DOI:** 10.1007/s12026-023-09436-7

**Published:** 2023-11-27

**Authors:** Weronika Ortmann, Anna Such, Elzbieta Kolaczkowska

**Affiliations:** 1 Laboratory of Experimental Hematology, Institute of Zoology and Biomedical Research, Jagiellonian University, Gronostajowa 9 Street, 30-387, Krakow, Poland; 2 Doctoral School of Exact and Natural Sciences, Jagiellonian University, Krakow, Poland

**Keywords:** Extracellular vesicles, Systemic inflammation, Endotoxemia, Ceruloplasmin, RAW 264.7 cell line

## Abstract

Microparticles (MPs) packaged with numerous bioactive molecules are essential vehicles in cellular communication in various pathological conditions, including systemic inflammation, Whereas MPs are studied mostly upon isolation, their detection *in vivo* is limited. Impact of MPs might depend on target cell type and cargo they carry; thus herein, we aimed at verifying MPs’ impact on macrophages. Unlike neutrophils, monocytes/macrophages are rather inactive during sepsis, and we hypothesized this might be at least partially controlled by MPs. For the above reasons, we focused on the detection of MPs with intravital microscopy (IVM) and report the presence of putative neutrophil-derived MPs in the vasculature of cremaster muscle of endotoxemic mice. Subsequently, we characterized MPs isolated not only from their blood but also from the peritoneal cavity and observed differences in their size, concentration, and cargo. Such MPs were then used to study their impact on RAW 264.7 macrophage cell line performance (cell viability/activity, cytokines, oxygen, and nitrogen reactive species). Addition of MPs to macrophages with or without co-stimulation with lipopolysaccharide did not affect respiratory burst, somewhat decreased mitochondrial activity but increased inducible nitric oxide synthase (iNOS) expression, and NO production especially in case of plasma-derived MPs. The latter MPs carried more iNOS-controlling ceruloplasmin than those discharged into the peritoneal cavity. We conclude that MPs can be detected *in vivo* with IVM and their cellular origin identified. They are heterogeneous in nature depending on the site of their release. Consequently, microparticles released during systemic inflammation to various body compartments differentially affect macrophages.

**Supplementary Information:**

The online version contains supplementary material available at 10.1007/s12026-023-09436-7.

## Introduction

Cellular communication is a key factor in the course of inflammatory disorders [1]. It relays on either direct cell-to-cell interactions, soluble mediators released by involved cells or molecules delivered into recipient cells via extracellular vesicles (EVs) [1]. The outcome of the disease results from the sum of these interactions thus if they are overwhelming or not sufficient it might be inadequate to the challenge. This also applies to systemic inflammation or sepsis which is a syndrome of pathologic and biochemical abnormalities caused by infection making it a leading cause of mortality and critical illness worldwide [2]. In particular, sepsis is a multifaceted host response to the infecting pathogen, involving both pro- and anti-inflammatory responses, and it may be significantly amplified by endogenous factors [3, 4]. The latter include extracellular vesicles consisting of exosomes, ectosomes (microparticles, MPs), and apoptotic bodies differing in size, mode of formation and biochemical composition. Exosomes are smaller (30-150 nm) and their formation is initiated by endocytosis [5], whereas MPs measure 0.1–1 μm [6], but sometimes can also reach larger size of up to 2 μm [7]. MPs are formed in a process of direct budding of the cell membrane thus they carry markers of the parental cell on their surface (e.g., in mice: Ly6G from neutrophils or F4/80 from monocytes/macrophages) [8–10]. EVs contain and transport numerous proteins, lipids, transcription factors [11, 12], cytokines [13], and genetic material in the form of mRNA, microRNA, and others [5]. They are detectable in blood [14, 15], local inflammatory exudate [16] and body fluids [17–19]. They are transported, even over long distances, to target cells on which they bind to adhesion proteins or lipids and are taken up and internalized via either membrane fusion, phagocytosis, or various endocytic pathways [20, 21]. EVs may act as either anti-inflammatory and pro-inflammatory factors, depending on the stimulus that generates them and the cell from which they are released. This is especially important as they are secreted not only in health but foremost in various pathological states, such as sepsis, and in large quantities [22].

One of the major immune cells engaged in sepsis is macrophages consisting of resident cells located in multiple tissues or inflammatory macrophages originating from monocytes chemotacting to the site of infection [23, 24]. During the early stages of any inflammation, macrophages usually display a pro-inflammatory phenotype but it can be modulated by EVs and other factors. For example, it was shown that MPs decrease neutrophil recruitment to the site of inflammation [25] and reduce numbers of macrophages during sepsis thereby leading to an increased dysfunction of the immune response [26]. The inflammatory phenotype of macrophages is characterized by the release of pro-inflammatory cytokines (e.g., interleukin 1β and IL-1β) and nitric oxide (NO) [27, 28]. The latter depends on the activity of inducible NO synthase (iNOS) which metabolizes L-arginine to NO and citrulline [29].

Herein, we aimed to verify if MPs collected from mice with experimental sepsis induced by lipopolysaccharide (LPS; endotoxemia) can change basic physiological characteristics of macrophages and their activity. Additionally, not only naïve macrophages were treated with MPs but also those simultaneously activated with LPS to mimic inflammatory conditions. MPs were collected from two body compartments, that is, blood and peritoneal cavity, to investigate if their effects on the tested cells depend on their source. We report that we isolated large quantities of MPs from both blood plasma and peritoneal exudate in the course of LPS-induced systemic inflammation in mice, but we were also able to visualize them *in vivo* in blood/circulation with intravital microscopy (IVM). We show that whereas isolated MPs enhance macrophage proliferation/viability, they decrease their mitochondrial activity whereas hardly impact respiratory burst. However, they increase iNOS expression (especially those originating from the peritoneal exudate) although without impacting NO production. The observed differences in impact of blood plasma- and peritoneal exudate-derived MPs may result from differences in bioactive cargo they carry during sepsis as in the former the content of an exemplary acute-phase reactant (ceruloplasmin, Cp) increases whereas in the latter it remains unaltered. Ceruloplasmin is one of the major multicopper-binding enzymes [30] which can also regulate the activity of iNOS and NO production [31]. Cp is synthesized principally by the liver but also by immune cells, i.e., monocytes, macrophages, and lymphocytes [32, 33]. An increased level of Cp is commonly detected in blood in the course of various pathological states, including sepsis [33–35].

In summary, depending on the source of MPs, they are characterized by different biological properties which are closely related to their cargo. This in turn differentially alters cellular responses induced by various MPs and as such suggests that microparticles originating from multiple body compartments should be studied simultaneously.

## Materials and methods

### Mice

C57BL/6J male mice were purchased from the Charles River Laboratories (Sulzfeld, Germany; via AnimaLab). Mice were used in experiments at the age of 8–12 weeks. Mice were housed in standard environmental conditions and were fed a commercial diet and had access to water (both *ad libitum).* All experimental animal protocols were approved by the Local Ethical Committee No. II in Krakow (294/2017 and 244/2019).

### Endotoxemic model

Mice were intraperitoneally (i.p.) injected with 1 mg/kg body weight (b.w.) of LPS (*Escherichia coli*, serotype 0111:B4; Sigma-Aldrich, Saint Louis, MO, USA) in saline to induce endotoxemia [36]. At selected time points, mice were subjected to intravital imaging, or their blood and peritoneal exudate were collected as detailed below.

### Sample collection for microparticle isolation

Mice were anesthetized with a mixture of ketamine hydrochloride (200 mg/kg b.w.; Biowet Pulawy, Pulawy, Poland) and xylazine hydrochloride (10 mg/kg b.w.; aniMedica, Südfeld, Germany) and subjected to blood and peritoneal exudate collection. Blood was collected by cardiac puncture in a heparinized syringe, and peritoneal fluid/exudate was collected by lavage of the peritoneal cavity with 1 mL of saline, and after 30 s of gentle manual massage, peritoneal fluid was retrieved. Samples were centrifuged at 400 × *g* and 1200 × *g* for 10 min at room temperature (RT) and then at 1500 × *g* for 15 min at RT for the retrieval of plasma and debris-free peritoneal fluid. Both blood and peritoneal exudate were collected at selected time-points (as indicated below and in figure legends; for *in vitro* RAW 264.7 cell stimulation MPs were collected at 8 h post-LPS administration) and were immediately subjected to the MP isolation procedure. Mice were euthanized by overdosing of ketamine/xylazine mixture.

### Microparticle isolation

MPs were isolated by sequential centrifugation. The first step was a negative isolation of leukocytes (400 × *g* and 1200 × *g*, 10 min at RT) and then of platelets (1500 × *g*, 15 min at RT) to remove cell debris but also apoptotic bodies. In the following step, the collected supernatant was pelleted in a centrifuge twice at 14,000 × *g* for 35 min at 4 °C (Sorvall WX+ Ultracentrifuge Series) [37].

### Nanoparticle tracking analysis

Before the second centrifugation as in *Microparticle Isolation*, the MP pellet was washed with sterile 1x phosphate-buffered saline (PBS) and was analyzed with Nanoparticle Tracking Analysis (NTA) using the NanoSight system–LM10HS microscope equipped with the LM14 488 nm laser module (Malvern Instruments Ltd., Malvern UK). The size, distribution, and concentration of blood plasma- and peritoneal exudate-derived MPs were determined by NTA which track in real time each particle individually in liquid suspension, using both Brownian motion and light scattering. For this purpose, samples with MPs were diluted 500 times in prefiltered (0.2 μm) sterile PBS to the total volume of 1 mL. Subsequently, the solution was placed into an insulin syringe and loaded into the sample chamber. The MP size and concentration measurements were taken from 60-s video duration, captured by the sCMOS camera and calculated using the NanoSight NTA 2.3 analytical software. The samples were analyzed with the same settings.

### Flow cytometry analysis

Employing flow cytometry (FACSCanto, BD Biosciences Immunocytometry Systems, San Jose, CA, USA), MPs isolated from blood, and peritoneal fluid were gated as neutrophil-derived Ly6G^+^ MPs (PE anti-mouse Ly6G, 1A8, BioLegend, San Diego, CA, USA), and monocyte/macrophage-derived F4/80^+^ MPs (PE anti-mouse F4/80, BM8; eBioscience, San Diego, CA, USA). Microparticles were analyzed using FACSDiva v8.0.1 software (BD Biosciences). The corresponding isotype control rat IgG2a, κ (PE IgG2a antibody, κ; RTK2758; BioLegend, San Diego, CA, USA), was analyzed in parallel.

### Preparation of cremaster muscle for intravital microscopy (IVM)

Prior to IVM, healthy and endotoxemic mice (8 h post LPS-administration) were anesthetized with a mixture of ketamine and xylazine hydrochloride as described above. Subsequently, cannulation of the right jugular vein was performed to administrate anesthetics/antibodies. Preparation of the cremaster muscle for IVM was performed as previously described [8, 38]. Briefly, mice were placed on the Plexiglas board dedicated for cremaster muscle visualization. A small piece of the scrotum was cut to expose the left testis from which cremaster muscle was carefully extracted. Then, the muscle was cleaned of connective tissue and separated from the testis by thermal cutting (with a cautery) of the thin ligaments connecting the muscle with the testis. Visualized cremaster muscle was placed on a previously prepared optically clear board. The testis and epididymis were put back inside, into the abdominal cavity.

### Visualization of microparticles and leukocytes in the cremaster vasculature with IVM

Monocytes/monocyte-derived MPs were visualized with PE anti-F4/80 (1.2 μg/mouse, clone BM8; eBioscience, San Diego, CA, USA), and neutrophils and neutrophil-derived MPs were stained with anti-mouse Ly6G antibodies (1.2 μg/mouse; PE or Brilliant Violet 421 anti-Ly6G, clone 1A8, BioLegend, San Diego, CA, USA). Endothelial cells were stained with anti-CD31 antibodies (5 μg/mouse; Alexa Fluor 647 anti-CD31, clone 390, BioLegend, San Diego, CA, USA). Cells and MPs were visualized per 20× field of view (FOV).

### Spinning disk confocal intravital microscopy (SD-IVM)

The cremaster muscle was imaged with a ZEISS Axio Examiner.Z1 upright microscope equipped with a metal halide light source (AMH-200-F6S; Andor, Oxford Instruments, Abingdon, UK) with motorized 6 position excitation filter wheel and laser-free confocal spinning disk device (DSD2; Andor, Oxford Instruments, Abingdon, UK) with ZEISS EC Plan-NEOFLUAR 10×/0.3 and/or ZEISS EC Plan-NEOFLUAR 20×/0.5 air objective. The following filters were used, four excitation filters (DAPI: 390/40 nm; GFP: 482/18 nm; RFP: 561/14 nm; Cy5: 640/14 nm), and appropriate emission filters (DAPI: 452/45 nm; GFP: 525/45 nm; RFP: 609/54 nm; Cy5: 676/29 nm). For fluorescence detection, the 5.5-megapixel sCMOS camera (Zyla 5.5; Andor, Oxford Instruments, Abingdon, UK) was used and the iQ 3.6.1 acquisition software (Andor, Oxford Instruments, Abingdon, UK) to drive the microscope.

### Cell culture of RAW 264.7 macrophages

RAW 264.7 mouse macrophage cell line (American Type Culture Collection; Manassas, VA, USA) was used in the study. Cells were cultured in RPMI 1640 medium (Lonza, Switzerland), supplemented with 10% fetal bovine serum (FBS; Biowest, USA), 2% penicillin/streptomycin and 1% L-glutamine (Sigma-Aldrich, Germany), at 37 °C in a 5% CO_2_ incubator (Thermo electron, USA). Cells were subcultivated in T-75 cell culture flasks (Nunc, Denmark). Cells used in the experiments were collected between 4 and 15 passages. Cells from passages 4–8 were defined as originating from “low passages” and cells from > 10 passages as “high passages.” The majority of studies (if not clearly stated otherwise) was performed on cells from “low passages”. All cell culturing was performed in a laminar chamber (ESCO, Singapore). Once the cells were 80–85% confluent, they were detached using mechanical scrapers (Biologix, USA), gently re-suspended, and then placed in a sterile 15-mL centrifuge tube (NEST, China). Next, they were centrifuged at 1500 rpm for 10 min (MPW, Poland). The supernatant was removed, and the cells were re-suspended in 1 mL of fresh complete medium. Cell counts were performed using a Bürker hemocytometer. Additionally, cell viability was determined by Trypan blue assay (Sigma-Aldrich, Germany). The cells were then subcultured to 96-well tissue culture plates (NEST, China), adding 1 × 10^5^ cells per well and then incubated at 37 °C in 5% CO_2_ for 30 min to let them adhere.

### Macrophage stimulation

RAW 264.7 macrophages were stimulated with LPS and/or MPs. LPS-treated cultures served as a positive control [39] and some cells were left unstimulated (negative control). Experimental groups consisted of cells stimulated with MPs isolated from blood plasma or peritoneal exudate that were added in two concentrations: 8 × 10^6^ and 16 × 10^6^ per well. Numbers of MPs were established by NTA as described above. Some of the cells were additionally stimulated by LPS at a concentration of 10 μg/mL. The cells were then incubated overnight at 37 °C in 5% CO_2_ until further analysis. In some experiments, cells were incubated for 1, 3, 6, and 9 hours instead of 18 hours (overnight) wherever indicated. In some studies, prior (30 min) or after (30 min) LPS and MP stimulation following inhibitors were added: NG-nitro-L-arginine methyl ester (L-NAME; NOS inhibitor) and N-(3-aminomethyl)-benzylacetamidine (1400W; specific iNOS inhibitor) in concentration of 2 mM and 50 μM, respectively. After overnight stimulation, NO was measured in supernatants and fixed cells were subjected to immunocytochemistry to detect iNOS as described below.

### Crystal violet (CV) assay

Macrophage numbers were estimated by the crystal violet (CV) assay that indirectly verifies cell numbers and viability by evaluation of cell adherence to culture plates [40]. After overnight incubation with LPS and/or MPs, culture medium was carefully removed from the wells. Next cells were fixed in absolute methanol (Chempur, Poland) for 10 minutes at RT. Methanol was then removed and crystal violet solution (Sigma-Aldrich, Germany) was added to each well (25 mg CV was dissolved in 5 mL 20% methanol). Plates were incubated for 5 min, washed twice in tap water to remove the unbound dye and then dried. To extract CV from the cells, 100% methanol was added to wells. Plates were agitated for 10 min, and absorbance was measured at 570 nm in a microplate reader (Tecan, Infinity F200 Pro, Männedorf, Switzerland).

### Presto Blue viability assay

After overnight stimulation with LPS and/or MPs, proliferation/viability of RAW 264.7 macrophages was evaluated by the PrestoBlue assay (BioVision, USA) [41]. Briefly, at the end of the incubation time, PrestoBlue reagent was added to each well and then plates were incubated for another 30 min at 37 °C in 5% CO_2_ in darkness. The fluorescence was measured at 610 nm (excitation 535 nm, emission 595 nm) in a microplate reader.

### MTT assay

To determine mitochondrial activity of RAW 264.7 macrophages, 3-(4,5-dimethylthiazol-2-yl)-2,5-diphenyltetrazolium bromide solution (MTT; Sigma-Aldrich, Germany) was added to each well in a final concentration of 5 mg/mL after overnight incubation with LPS and/or MPs. The cells were incubated for 3 h at 37 °C and 5% CO_2_. The medium was then removed, and the formazan precipitate was solubilized in acidic isopropanol (Chempur, Poland). The absorbance was measured at 570 nm in a microplate reader.

### Nitric oxide (NO) production assay

The generation of nitric oxide was measured with the Griess assay [42]. The RAW 264.7 cells were incubated in 96-well plates overnight in a presence of LPS and/or MPs as described above. After this time, cell culture supernatants were collected and frozen prior to further analyses. To run the assay, the samples were thawed and 50 μL was mixed with equal volume (25 μL each) of Griess reagents A (5% phosphoric acid containing 1% sulfanilamide) and B (0.1% solution of N-(1-naphthyl)ethylenediamine in distilled water (dH_2_O)); both from Sigma-Aldrich (Saint Louis, MO, USA). After 10 min at RT, the absorbance was measured at the wavelength 570 nm using a microplate reader.

### NBT assay: respiratory burst measurement

The intracellular reactive oxygen species (ROS) production by RAW 264.7 macrophages was determined using 3,3′-(3,3′-dimethoxy-4,4′-biphenylene)bis[2-(4-nitrophenyl)-5-phenyl-2H-tetrazolium chloride] (NBT) reduction assay [43]. Phorbol myristate acetate (PMA) was used as a positive control as it induces ROS synthesis [44]. One hour before the end of the overnight incubation with LPS and/or MPs, 50 nM PMA (Sigma-Aldrich, Germany) was added into some wells (RPMI 1640 was added to PMA-non-stimulated wells). Plates were incubated for 1 h and then NBT solution (10 mg/mL; Sigma Aldrich, Germany) was added, and the plates were further incubated for 1 h at 37 °C and 5% CO_2_. The supernatants were removed, and the cells were fixed for 15 min in absolute methanol, washed twice with 70% methanol, then dried. The formazan deposits were solubilized in 120 mL of 2 M KOH and 140 mL dimethyl sulfoxide (DMSO; both from Sigma-Aldrich, Germany). After homogenization of the well content, the absorbance was read at 590 nm using a microplate reader.

### Immunocytochemistry: iNOS expression

In some experiments, prior to seeding RAW 264.7 macrophages in 96-well plates, coverglasses were inserted into the wells. Then the cells were added, and they were let to adhere for 30 min. Subsequently they were stimulated with LPS and/or MPs as described above. After the overnight incubation, supernatants were carefully removed and the cells were fixed in a sequence of 1%, 2%, and 3% paraformaldehyde in PBS for 2, 10, and 20 min, respectively, and then washed in PBS for 5 min [45]. Prior to immunocytochemistry, for cell membrane permeabilization, the cells were washed for 5 min in TBS solution which contained Triton X-100 (Sigma-Aldrich, Germany), Na_2_HPO_4_ × 12H_2_O, Na_2_HPO_4_ × 1H_2_O (both from Avantor Performance Materials Poland), bovine serum albumin (BSA; Sigma-Aldrich, Germany), NaCl (Polfa–Lublin S.A., Poland), and dH_2_O. After washing, the coverglasses were incubated in blocking solution, namely 3% BSA in PBS, for 45 min at RT. Next the cells were labelled with rabbit monoclonal anti-iNOS antibodies (EPR16635 clone; Abcam, Cambridge, UK) diluted 1:200 in 1% BSA/PBS and incubated overnight at 4 °C in a humid chamber. The coverglasses were then washed twice in PBS and further incubated with secondary Cy3-conjugated goat anti-rabbit antibodies (Jackson Immunoresearch Laboratories, Ely, UK) diluted 1:300 in 1% BSA/PBS for 1 h at RT. Following the incubation, the slides were washed in PBS for 5 min and counterstained with 5 μM Sytox green (Invitrogen, Carlsbad, CA, USA) to visualize nuclei. After 5 min of staining, the coverglasses were washed in PBS and mounted with VECTASHIELD Mounting Medium (Vector Laboratories, Burlingame, CA, USA). Fluorescent signal was detected with a ZEISS Axio Examiner.Z1 upright microscope equipped with confocal spinning disk device DSD2 (Andor, Oxford Instruments, Abingdon, UK; the microscope details as above). The percentage of iNOS positive cells was estimated with ImageJ software (US National Institutes of Health, Bethesda, MD, USA).

### Cytokine measurement

The cell culture supernatant content of mouse IL-1β and IL-6 was measured by Mouse IL-1β ELISA Ready-SET-Go (Affymetrix/ThermoFisher Scientific, Waltham, MA, USA) and Mouse IL-6 ELISA Ready-SET-Go (eBioscience/ThermoFisher Scientific, Waltham, MA, USA). The assays were carried out as indicated by the manufacturers.

### Western blot analysis

Freshly isolated MPs were lysed in RIPA buffer (Thermo Fisher Scientific, Waltham, MA, USA) in the presence of the protease inhibitor cocktail (Thermo Fisher Scientific, Waltham, MA, USA). The prepared samples were subjected to thermal denaturation (5 min, 95 °C) by placing them on a thermoblock (OHAUS, Poland) and then mechanical disruption using a sonicator (Bandelin, Germany). Protein concentration in samples was determined using the Bradford method. For this purpose, concentrated Bradford reagent (Bio-Rad Protein Assay Dye Reagent Concentrate; Bio-Rad; USA) was used, which, after appropriate (5x) dilution in dH_2_O, was added to the samples and to the standard curve (BSA solution). Absorbance was measured at the 595 nm wavelength, and the ELx808 ELISA plate reader and the KC Junior software (BioTek Instruments, Winooski, VT, USA) were used. Then the samples (proteins) were normalized for further analysis. Commercially purchased 4-20% Mini-PROTEAN® TGXTM Precast Protein Gel (BioRad Laboratories, Hercules, CA, USA) were used for protein separation in a Mini-PROTEAN TetraSystem vertical electrophoresis apparatus (BioRad Laboratories, Hercules, CA, USA). The transfer of separated proteins was performed using the Trans-Blot Turbo Transfer System (BioRad Laboratories, Hercules, CA, USA) in commercially purchased transfer packets Trans-Blot Turbo Mini 0.2 μm PVDF Transfer Packs (BioRad Laboratories, Hercules, CA, USA), and the manufacturer’s instructions were followed. After transfer, the blots were placed in blocking buffer (5% BSA [w/v] in 0.02 M Tris-buffered saline containing 0.1% Tween 20 (TBST) [v/v]) for 1.5 h (at RT) to block non-specific antibody binding to the membrane. Then, the membranes were probed overnight at 4 °C with primary recombinant rabbit monoclonal antibody (anti-ceruloplasmin, ARC5018-06-01 clone; Thermo Fisher Scientific, Waltham, MA, USA) 1:1000 at 5% BSA/TBST [w/v]). Next, the membranes were washed with TBST (3 times for 10 min) and then incubated for 1 h (at RT) with a horseradish peroxidase (HRP)-conjugated secondary antibody, anti-goat (Cell Signalling Technology, Massachusetts, DA, USA) 1:1000 at 5% BSA/TBST [w/v]. An anti-β-actin monoclonal antibody (13E5 clone; Cell Signalling Technology, Massachusetts, DA, USA) 1:1000 at 5% BSA/TBST [w/v] was used as a control. After that, the blots were washed in TBST and 0.02 M Tris-buffered saline (TBS; twice and once, respectively, for each 10 min). Clarity Max Western ECL Substrate (BioRad Laboratories, Hercules, CA, USA) was used to detect chemiluminescence signal which was visualized by the GeneGnome Bio Imaging System (Syngene, Cambridge, UK). Densitometric analysis of the visible bands was performed with ImageJ software (US National Institutes of Health, Bethesda, MD, USA).

### Statistical analyses

All data are presented as mean values ± SD. Data were compared either by unpaired two tailed Student’s *t*-test (to evaluate the means of two populations) or one-way analysis of variance (ANOVA; to compare the means of more than two groups) with Bonferroni’s multiple comparisons *post hoc* test. Statistical significance was set at *P* < 0.05.

## Results

### Microparticles can be visualized in vivo in the vasculature of endotoxemic mice

**Fig. 1:**
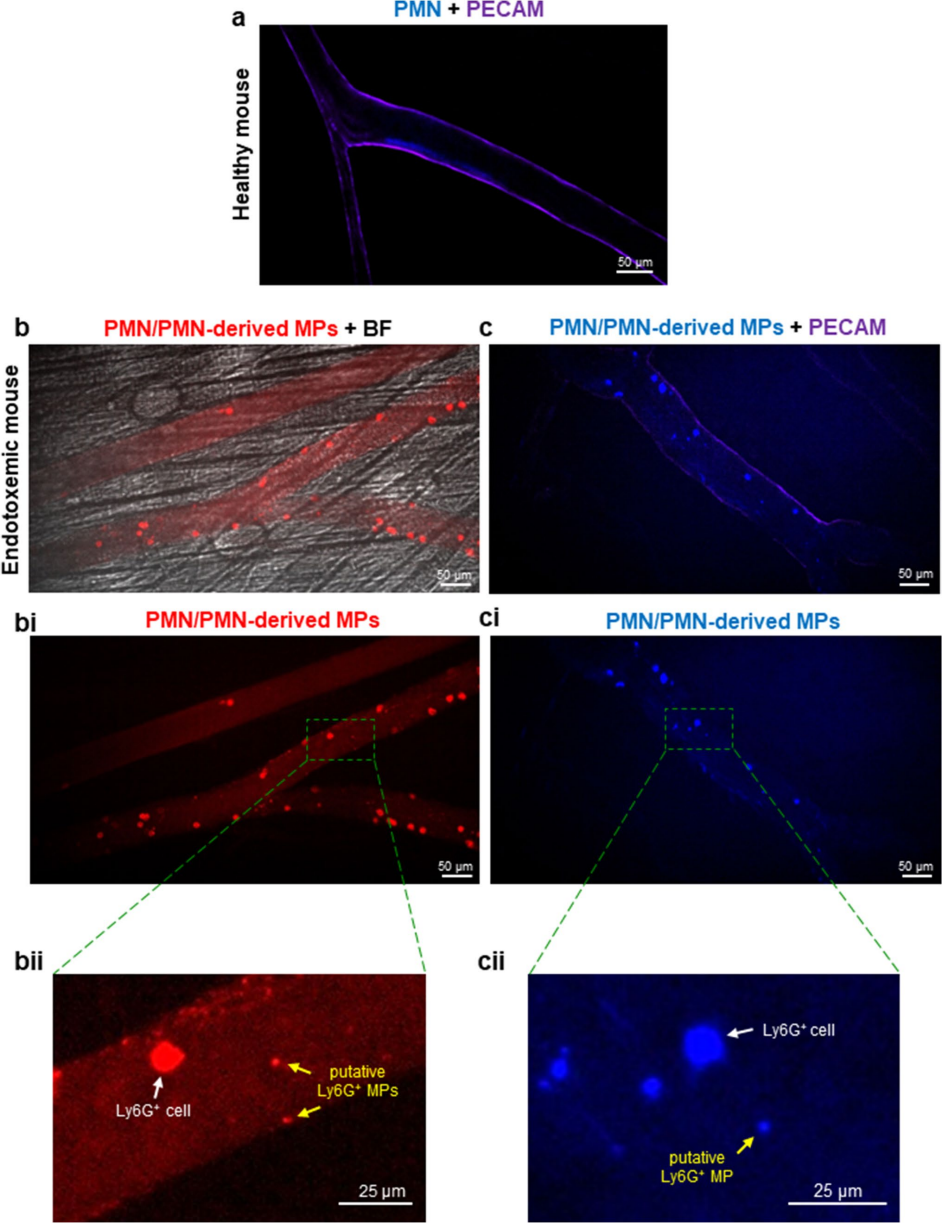
Presence of neutrophils (PMNs) and putative neutrophil-derived microparticles (MPs) in the vasculature of the cremaster muscle of healthy and endotoxemic mice. C57BL/6J healthy mice and mice with LPS-induced endotoxemia (1 mg/kg b.w.; 8 h post LPS inoculation) were subjected to *in vivo* imaging with Spinning Disk Confocal Intravital Microscopy (SD-IVM). **a** A representative image of the cremaster muscle vasculature in a healthy mouse (PECAM, violet) and **b, c** in mice with 8-hour endotoxemia. In **b**, bright field (BF) is shown against the confocal channel (Ly6G, red) to visualize localization of blood vessels, and in **c**, the PECAM staining (violet) marks the vascular wall. **bii, cii** are enlarged fragments of **bi, ci** in which only Ly6G^+^ neutrophils are visible upon staining with **b** PE- or **c** Brilliant Violet 421-conjugated anti-Ly6G antibodies (red and blue, respectively). Along neutrophils, putative MPs are visible. The scale bar indicates 25 or 50 μm as labelled on each image.

To identify and visualize MPs *in vivo* in the vasculature of the cremaster muscle intravital microscopy was used. We detected putative monocyte- (not show) and neutrophil-derived MPs (Fig. 1) with antibodies recognizing their respective surface markers, F4/80 and Ly6G. Moreover, we were able to image release of Ly6G^+^ MPs in real time (not shown). We did not observe presence of any MPs adhering to endothelium in healthy mice, whereas following LPS treatment neutrophils started to adhere to the endothelium of the cremaster muscle vasculature and we observed presence of structures resembling MPs of various sizes (Fig. 1bii, cii). In contrast, we observed only a few monocytes and monocyte-derived MPs in contact with endothelium at this time point of endotoxemia (not shown).

### MPs isolated from blood and peritoneum differ by size

**Fig. 2:**
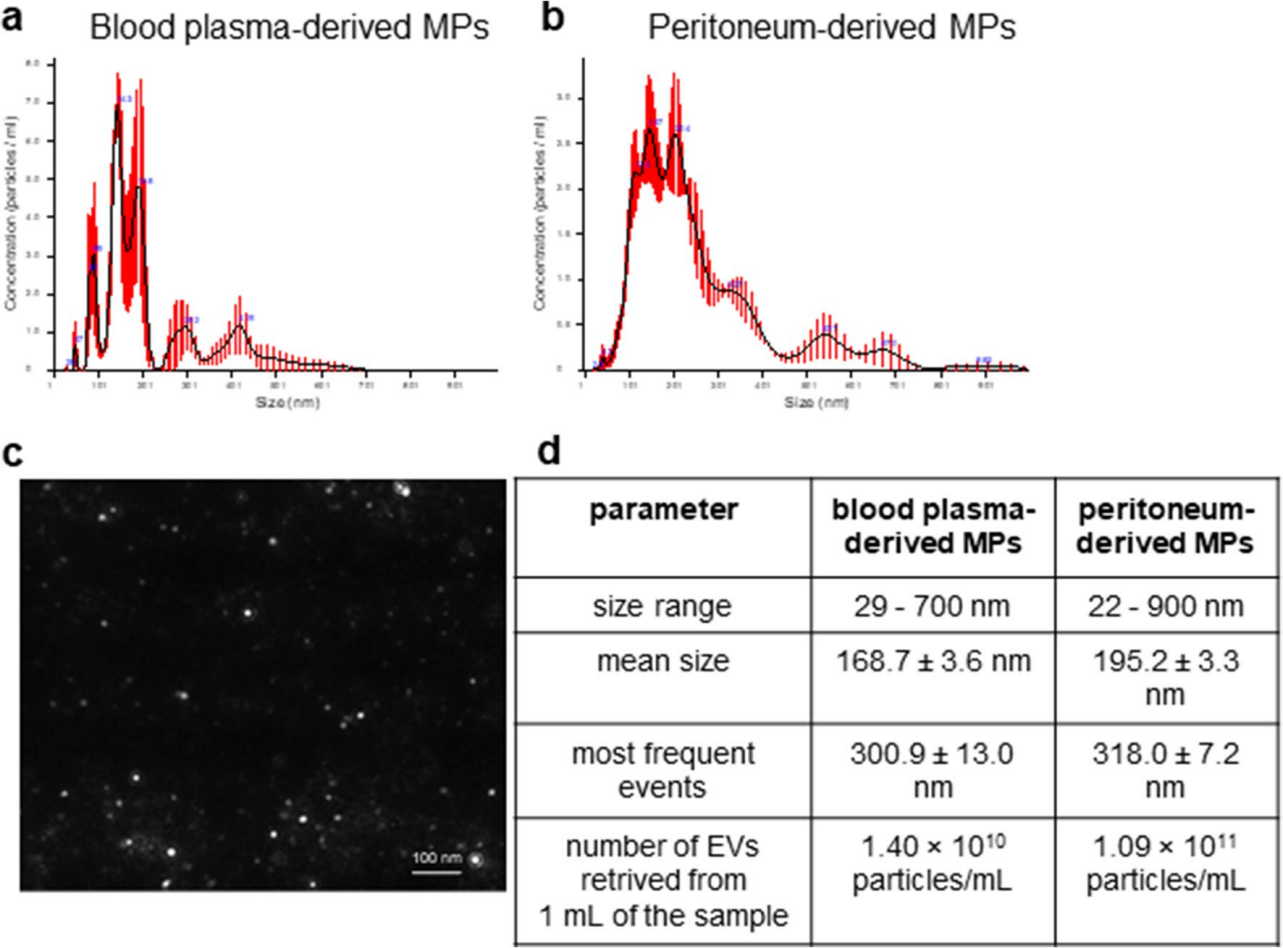
The basic characteristics of microparticles (MPs) acquired by Nanoparticle Tracking Analysis (NTA). Representative size frequency of MPs collected from **a** blood plasma and **b** peritoneum at 8 h of lipopolysaccharide-induced systemic inflammation (1 mg/kg b.w.) in C57BL/6J mice. **c** Exemplary image illustrating plot of blood plasma-derived MP obtained from endotoxemic mice. **d** Summary of the main features of blood plasma and peritoneum-derived MPs. The scale bar indicates 100 nm.

NTA analysis showed different distribution of MPs from blood plasma and peritoneal exudate, both in terms of size and concentration (heterogeneity; Fig. 2a, b). The size of plasma-derived MPs ranged from app. 29 to 700 nm (Fig. 2a; image illustrating their size—Fig. 2c) while in the peritoneum-derived MPs from 22 nm to 900 nm (Fig. 2b). The mean size of the former was 168.7 ± 3.6 nm, making them smaller than the peritoneum-derived MPs (195.2 ± 3.3 nm) (Fig. 2d). The most frequent events were around 300.9 ± 13.0 nm (blood plasma-derived EVs) and around 318.0 ± 7.2 nm (peritoneum-derived EVs) (Fig. 2d), and the smallest around 80.0 ± 2.0 nm (blood plasma-derived EVs) and 91.8 ± 3.7 nm (peritoneum-derived EVs) (data not shown). The smallest particles under 100 nm (app. 10% of the entire particle populations) might represent exosomes. Moreover, obtained MPs not only differ in size, but also in concentration. NTA analysis showed smaller number of plasma MPs compared to MPs obtained from the peritoneum (1.40 × 10^10^ particles/mL *vs.* 1.09 × 10^11^ particles/mL, respectively) (Fig 2d).

### Neutrophil and monocyte MPs in body fluids

Using flow cytometry, the MPs of neutrophil (Ly6G^+^) and monocyte/macrophage (F4/80^+^) origin present in blood plasma and peritoneal fluid were estimated during endotoxemia (8 h; Supplementary Fig. S1). MPs of monocyte/macrophage origin dominated over neutrophil-derived MPs in both body fluids (Supplementary Fig. S1).

### MPs of either origin increase macrophage proliferation and impact their mitochondrial activity

**Fig. 3:**
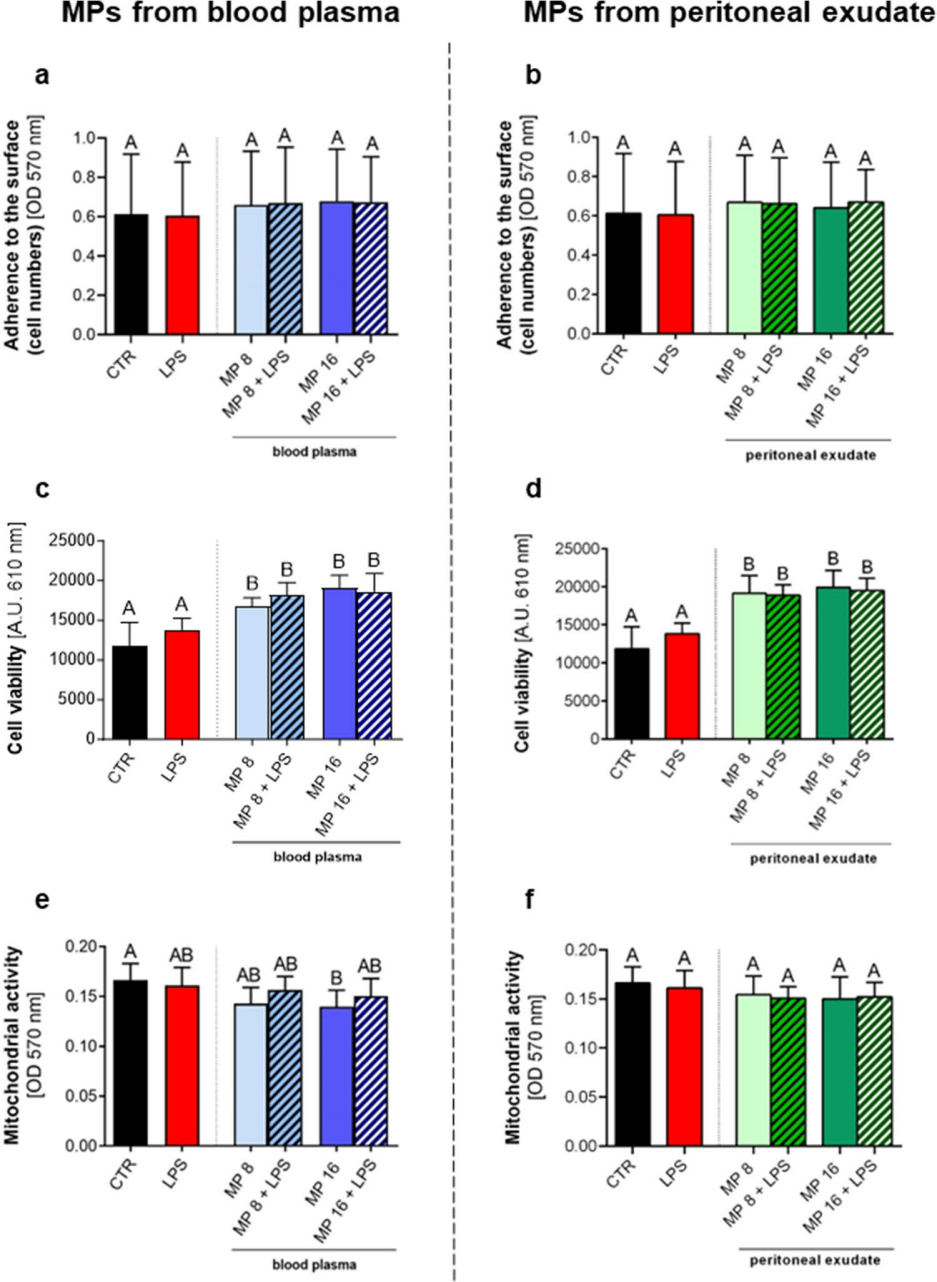
Effects of microparticles (MPs) on the viability, adherence to the surface and mitochondrial activity of RAW 264.7 macrophages. The analyses were performed with crystal violet assay (CV), Presto Blue and MTT assay, respectively. Microparticles were isolated from blood plasma (left column) or from exudative/inflammatory fluid collected from the peritoneal cavity (right column) of mice with endotoxemia 8-h post lipopolysaccharide (LPS) injection. The following groups were studied: unstimulated cells (CTR), cells stimulated with 10 μg/mL of lipopolysaccharide (LPS), cells stimulated with MPs at concentration 8 × 10^6^ MP/10^5^ cells (MP 8), cells stimulated with MPs at concentration 8 × 10^6^ MP/10^5^ cells, and 10 μg/mL of LPS (MP 8 + LPS), cells stimulated with MPs at concentration 16 × 10^6^ MP/10^5^ cells (MP 16), and cells stimulated with MPs at concentration 16 × 10^6^ MP/10^5^ cells and 10 μg/mL of LPS (MP 16 + LPS). **a** Quantitative analysis of cell adherence to the surface stimulated with MPs from blood plasma and **b** from peritoneal exudate. Changes in RAW 264.7 viability under stimulation with MPs from **c** blood plasma and **d** from peritoneal exudate. Quantitative analysis of mitochondrial activity of macrophages stimulated with MPs from **e** blood plasma and **f** from peritoneal exudate. A.U., arbitrary unit; OD, optical density. The results are expressed as the mean values ± SD. Values significantly different (*p* ˂ 0.05) according to one-way ANOVA with Bonferroni’s multiple comparisons *post hoc* test are designated by letters (the same letters indicate no differences between groups; different letters indicate statistical differences).

**Fig. 4:**
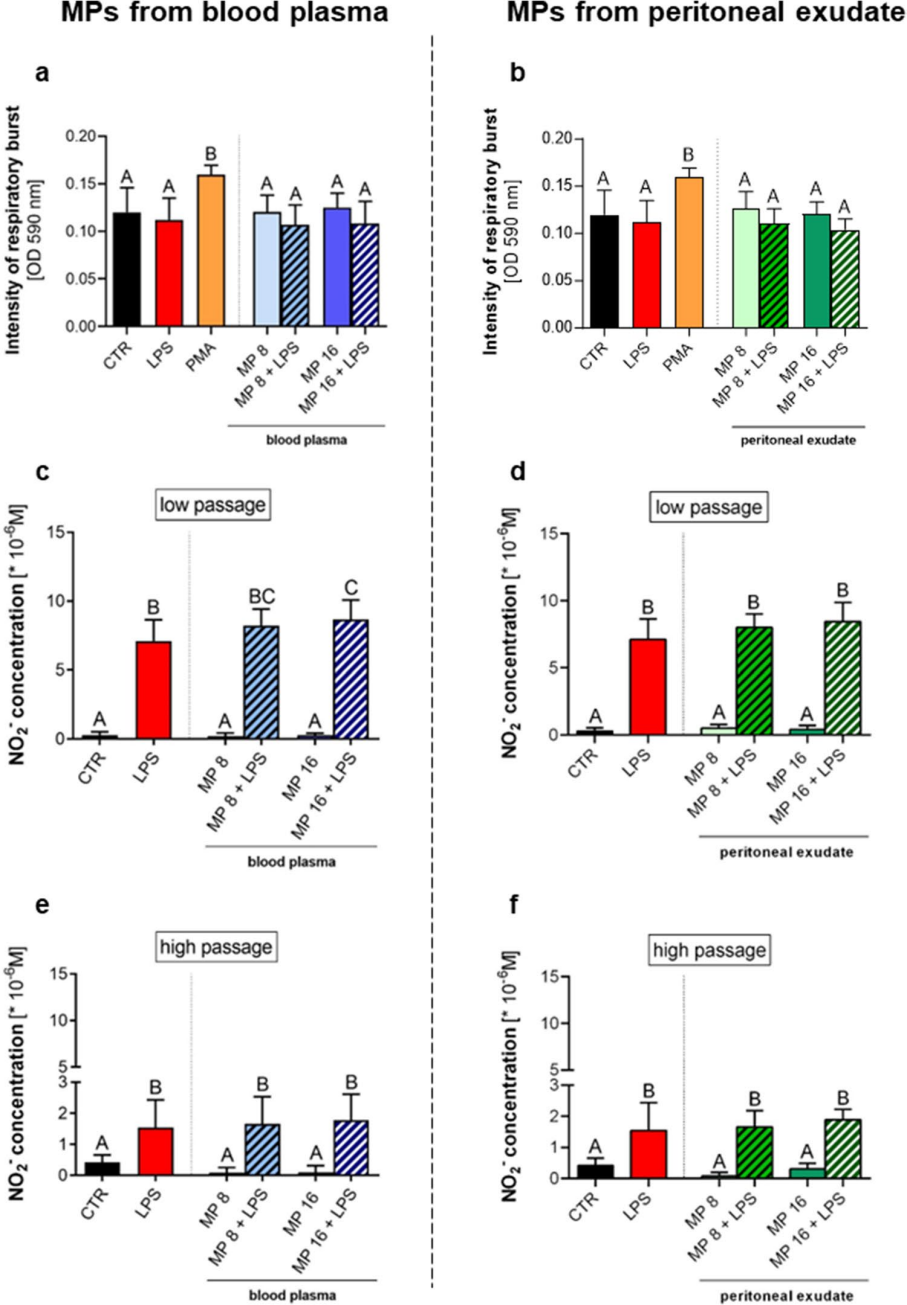
Effects of microparticles (MPs) on the reactive oxygen species (ROS) and nitric oxide (NO) production by RAW 264.7 macrophages were determined using NBT reduction and Griess assays, respectively. Microparticles were isolated from blood plasma (left column) or from exudative/inflammatory fluid collected from the peritoneal cavity (right column) of mice with endotoxemia 8 h post lipopolysaccharide (LPS) injection. The following groups were studied: unstimulated cells (CTR), cells stimulated with 10 μg/mL of lipopolysaccharide (LPS), cells stimulated with 50 nM of phorbol myristate acetate (PMA), cells stimulated with MPs at concentration 8 × 10^6^ MP/10^5^ cells (MP 8), cells stimulated with MPs at concentration 8 × 10^6^ MP/10^5^ cells and 10 μg/mL of LPS (MP 8 + LPS), cells stimulated with MPs at concentration 16 × 10^6^ MP/10^5^ cells (MP 16), and cells stimulated with MPs at concentration 16 × 10^6^ MP/10^5^ cells and 10 μg/mL of LPS (MP 16 + LPS). Changes in the intensity of respiratory burst of RAW 264.7 under stimulation with MPs from **a** blood plasma and **b** from the peritoneal exudate. Quantitative analysis of NO production by macrophages from **c** lower passages (4–8) and **e** from higher passages >10 stimulated with MPs from blood plasma. Evaluation of NO production levels by **d** low-passage RAW 264.7 cells under stimulation with MPs from the peritoneal cavity and **f** by high-passage cells. OD, optical density. The results are expressed as the mean values ± SD. Values significantly different (*p* ˂ 0.05) according to one-way ANOVA with Bonferroni’s multiple comparisons *post hoc* test are designated by letters (the same letters indicate no differences between groups; different letters indicate statistical differences).

**Fig. 5:**
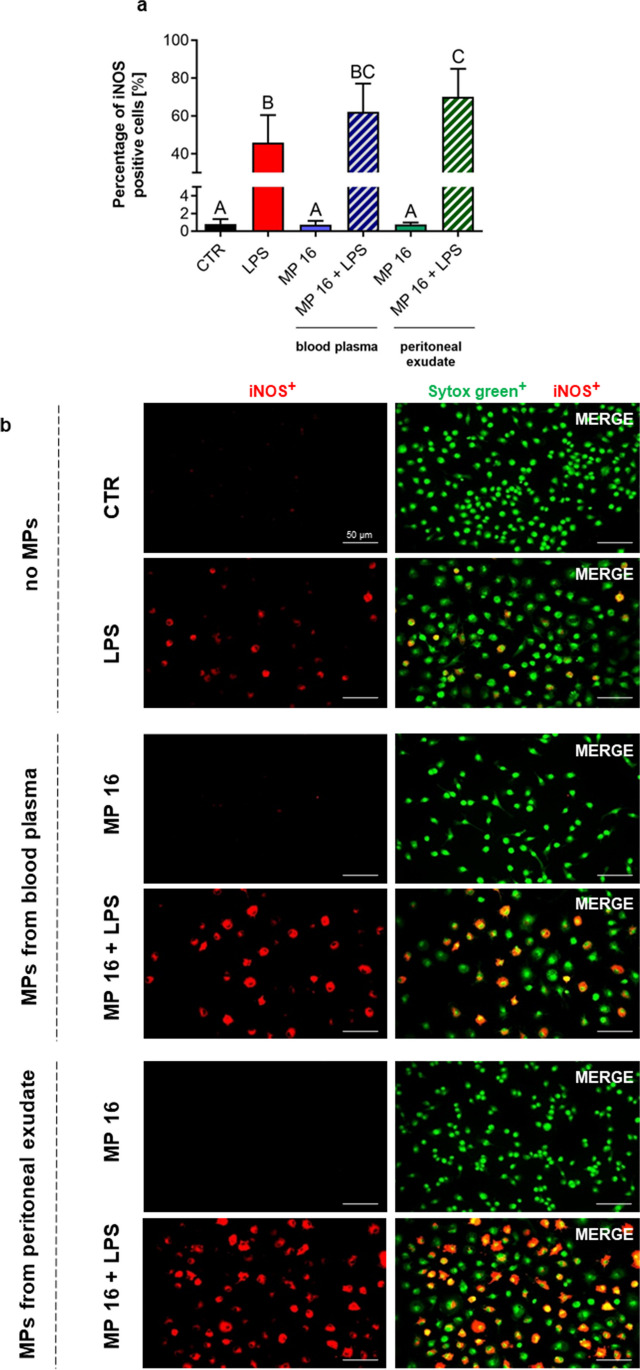
Expression of inducible nitric oxide synthase (iNOS) in macrophages upon microparticle (MP) stimulation. Microparticles were isolated from blood plasma or from exudative/inflammatory fluid collected from the peritoneal cavity of mice with endotoxemia 8 h post lipopolysaccharide (LPS) injection. **a** Quantification of iNOS expression by RAW 264.7 cells: percentage (%) of iNOS-positive cells. Following groups were studied: unstimulated cells (CTR), cells stimulated with 10 μg/mL of lipopolysaccharide (LPS), cells stimulated with MPs at concentration 8 × 10^6^ MP/10^5^ cells (MP 8), cells stimulated with MPs at concentration 8 × 10^6^ MP/10^5^ cells and 10 μg/mL of LPS (MP 8 + LPS), cells stimulated with MPs at concentration 16 × 10^6^ MP/10^5^ cells (MP 16), and cells stimulated with MPs at concentration 16 × 10^6^ MP/10^5^ cells and 10 μg/mL of LPS (MP 16 + LPS). The results are expressed as the mean values ± SD. Values significantly different (*p* ˂ 0.05) according to one-way ANOVA with Bonferroni’s multiple comparisons *post hoc* test are designated by letters (the same letter indicate no differences between groups, different letters indicate statistical differences). **(b)** Representative images of the expression of iNOS by macrophages upon MPs stimulation. iNOS positive cells (red) were visualized by co-staining of their nuclei with Sytox (green). The scale bar indicates 50 μm.

The effects of microparticles on the selected immunological parameters of RAW 264.7 macrophages were studied after an overnight stimulation (Figs. 3, 4, and 5). In order to determine whether the MPs impact macrophage numbers, the adherence of the cells to the surface was estimated with the crystal violet assay upon exposure of them to MPs isolated from blood plasma and peritoneal exudate (Fig. 3a, b, respectively). Compared to the control group stimulation of RAW 264.7 cells with either LPS, MPs (in different concentration), or MPs in conjunction with LPS resulted in no differences in numbers of adherent macrophages. Subsequently, cell proliferation/viability and mitochondrial activity was estimated by Presto Blue assay and MTT test, respectively (Fig. 3). Whereas Presto Blue assay revealed an increase in the cell proliferation/viability (Fig. 3c, d), the succinate dehydrogenase activity was decreased upon some treatments (Fig. 3e, f). In particular, when macrophages were treated only with higher concentration of MPs (from blood plasma) a decreased mitochondrial activity was observed (Fig. 3e) and a similar tendency was observed in other groups which received MPs (lower concentrations of MPs alone and both concentrations of MPs in conjunction with LPS; Fig. 3e).

### MPs do not alter the respiratory burst potential

The ability to generate ROS is an indicator of pro-inflammatory functions of macrophages [43]; thus, we performed the NBT assay. Only phorbol myristate acetate, serving as a positive control, increased the respiratory burst whereas neither MPs, LPS nor their combination influenced this parameter (Fig. 4a, b).

### MPs of various origin differentially regulate iNOS expression and nitric oxide release by macrophages

In light of the role of nitric oxide in pro-inflammatory function of macrophages, we measured NO production by cells exposed to MPs. Importantly, all the results presented so far were performed on RAW 264.7 cells obtained from “low passages” (4 to 8), but here we also tested cells from higher passages (> 10) (Fig. 4). Independently of the passage, increased NO production was observed upon LPS stimulation although in the case of the high-passaged cells, much smaller quantities of NO were released and MPs had no impact on this process (Fig. 4e, f). However, in the case of cells from the regular passages (“low passage”) addition of blood plasma MPs, especially in the higher concentration, increased NO release (Fig. 4c). To further investigate how NO release was changing over time, we also tested its synthesis at 1, 3, 6, and 9 hours post LPS but no further differences between groups were detected (data not shown) and its levels were highest 18–24 h post stimulation as shown in Fig. 4c–f. NO production depends on the expression of iNOS; thus, it was evaluated. As depicted on exemplary images presented in Fig. 5b, and then quantified (Fig. 5a), exposure of cells to MPs alone independently of their origin did not influence iNOS expression in comparison to control. However, upon LPS treatment, app. 50% of cells were iNOS-positive whereas following the addition of blood plasma MPs to LPS, about 60% of macrophages were expressing it. The highest percentage of iNOS-positive cells (app. 75%) was found in a group exposed to MPs isolated from the peritoneal exudate in conjunction with LPS (Fig. 5).

### MPs of either origin regulate pro-inflammatory cytokine production

**Fig. 6:**
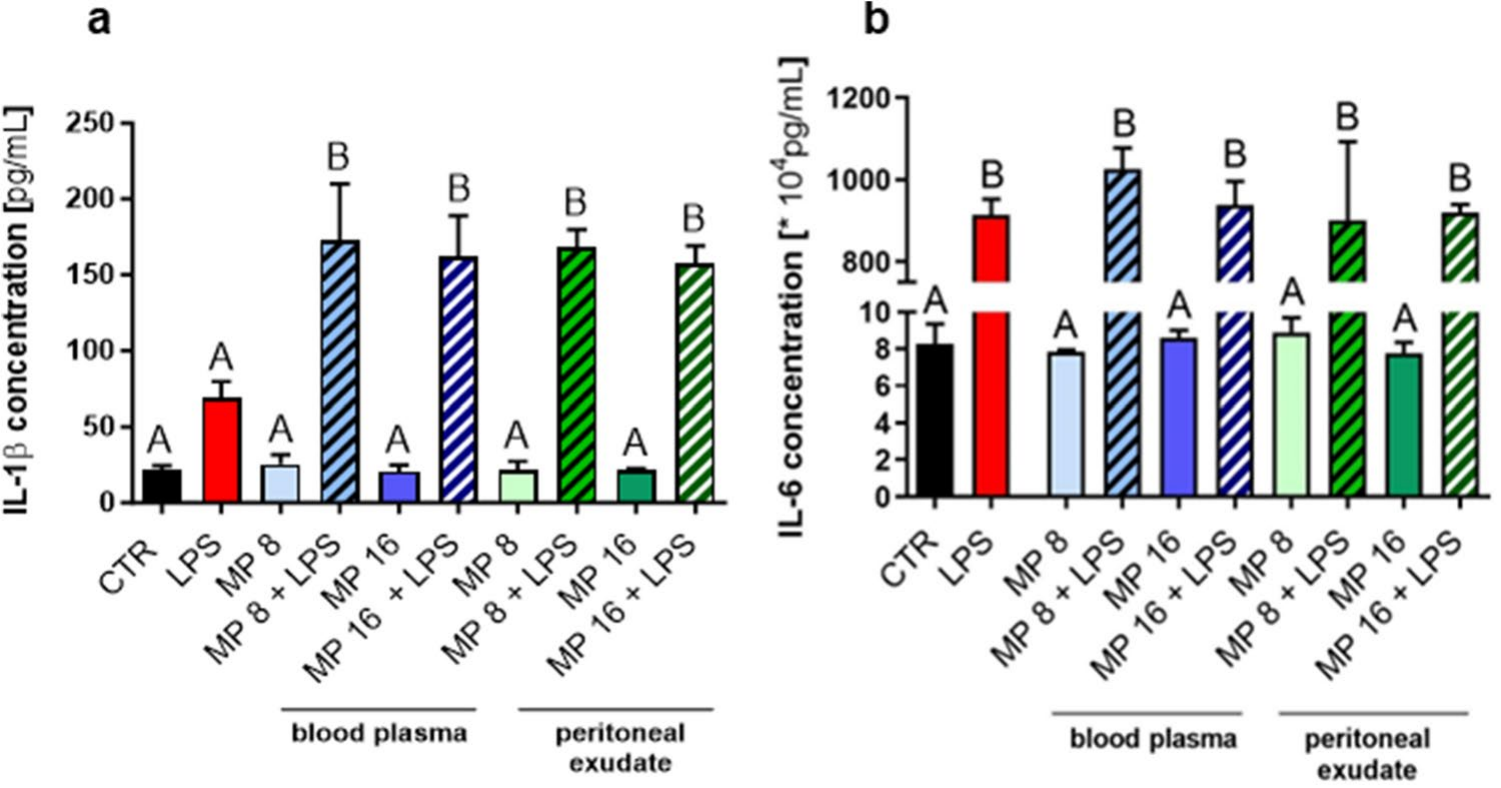
Effects of microparticles (MPs) stimulation on cytokine release by macrophages. Microparticles were isolated from blood plasma or from exudative/inflammatory fluid collected from the peritoneal cavity of mice with endotoxemia 8 h post lipopolysaccharide (LPS) injection. **a** IL-1β and **b** IL-6 release was estimated by ELISAs. Following groups were studied: unstimulated cells (CTR), cells stimulated with 10 μg/mL of lipopolysaccharide (LPS), cells stimulated with MPs at concentration 8 × 10^6^ MP/10^5^ cells (MP 8), cells stimulated with MPs at concentration 8 × 10^6^ MP/10^5^ cells and 10 μg/mL of LPS (MP 8 + LPS), cells stimulated with MPs at concentration 16 × 10^6^ MP/10^5^ cells (MP 16), and cells stimulated with MPs at concentration 16 × 10^6^ MP/10^5^ cells and 10 μg/mL of LPS (MP 16 + LPS). The results are expressed as the mean values ± SD. Values significantly different (*p* ˂ 0.05) according to one-way ANOVA with Bonferroni’s multiple comparisons *post hoc* test are designated by letters (the same letters indicate no differences between groups; different letters indicate statistical differences).

In order to verify if MP stimulation triggers pro-inflammatory activity of macrophages, IL-1β and IL-6 cytokine production was measured. There was indeed significantly higher IL-1β production by macrophages in groups exposed simultaneously to MPs and LPS, independently of MP origin or concentration, in comparison to LPS alone (Fig. 6a). In groups exposed to blood plasma MPs and peritoneal exudate MPs alone levels of IL-1β release were unchanged (Fig. 6a). The presence of microparticles alone (independently of their concentration and origin) did not stimulate IL-6 release by macrophages, only LPS alone and LPS co-applied with MPs of either origin and concentration increased IL-6 level (Fig. 6b).

### Blood plasma and peritoneal MPs contain ceruloplasmin in various proportions

**Fig. 7:**
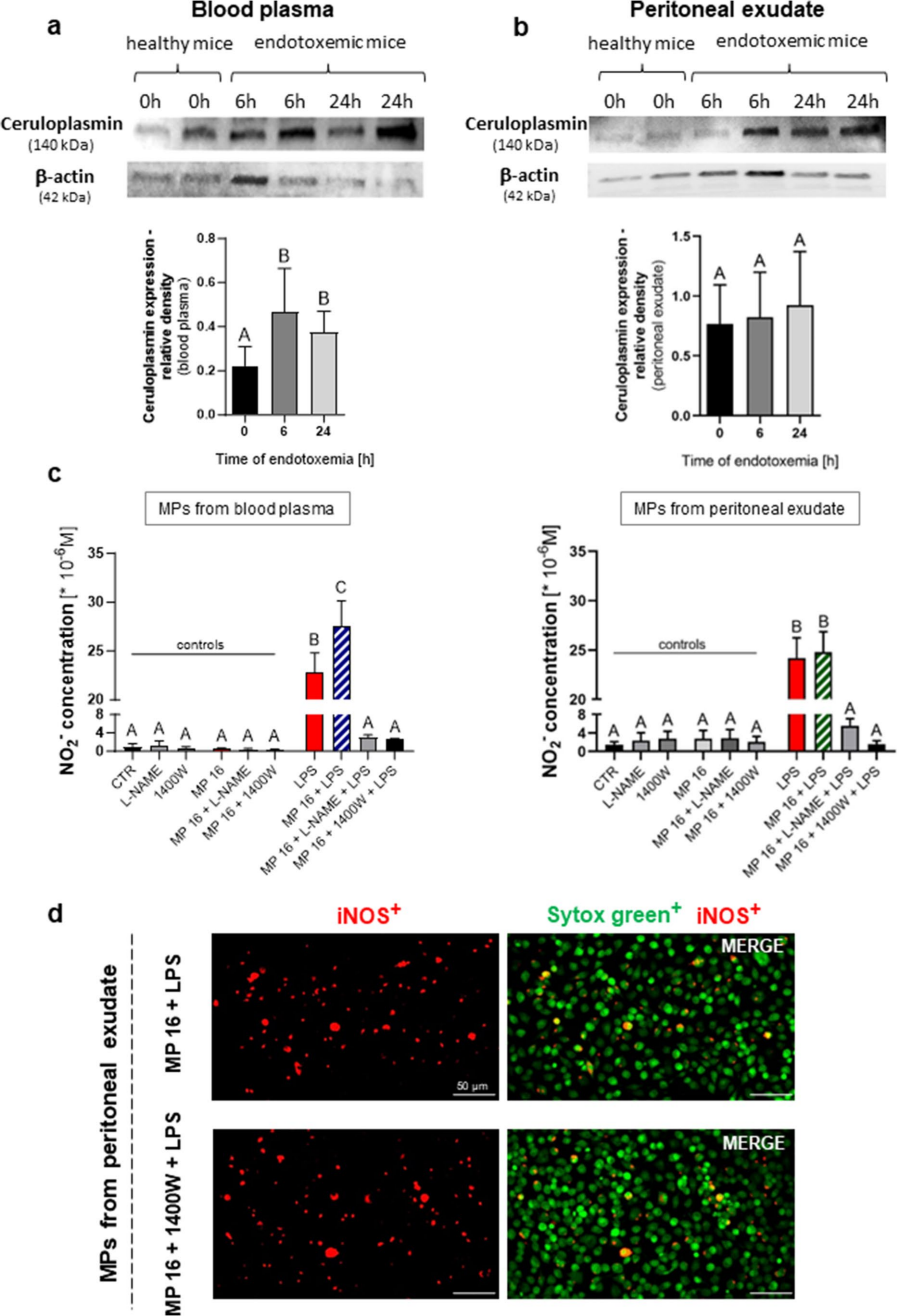
Western blot analysis of ceruloplasmin protein level in microparticles (MPs) and its effects on the inducible nitric oxide synthase (iNOS) activity and expression upon MP stimulation. MPs were obtained from blood plasma and from exudative/inflammatory fluid collected from the peritoneal cavity of C57BL/6J healthy mice and mice with LPS-induced endotoxemia (1 mg/kg b.w.; 6 and 24 h post injection). Representative (cropped) blot images and their graphs displaying the densitometric analysis (as calculated by ImageJ v1.53a software) of ceruloplasmin in MP obtained from **a** blood plasma and **b** peritoneal exudate. β-Actin was used as loading control. **c** Quantitative analysis of NO production by RAW 264.7 cells from lower passages (4–8) stimulated with MPs isolated from blood plasma and exudative/inflammatory fluid after 8 h of LPS-induced endotoxemia. Following groups were studied: control groups (unstimulated cells, CTR), and the groups (unstimulated cells, and cells stimulated with MPs (16 × 10^6^ MP/10^5^) in combination with/without 10 μg/mL of LPS) in the presence of NOS inhibitors (2mM of L-NAME and 50μM of 1400W). The results are expressed as the mean values ± SD. Values significantly different (*p* ˂ 0.05) according to one-way ANOVA with Bonferroni’s multiple comparisons post hoc test are designated by letters (the same letters indicate no differences between groups; different letters indicate statistical differences). **d** Representative images of the expression of iNOS by macrophages upon MPs stimulation. iNOS positive cells (red) were visualized by co-staining of their nuclei with Sytox (green). The scale bar indicates 50 μm.

Effects of MPs depend on the cargo they carry; thus, we tested the content of MPs originating from healthy mice (designated as 0 h of sepsis) and collected at 6 and then 24 h post *in vivo* LPS application. Western blot analysis was used to identify the presence of an acute-phase reactant and one of the major copper-binding proteins—ceruloplasmin (Fig. 7). Ceruloplasmin was detected in both blood plasma (Fig. 7a) and peritoneal exudate (Fig. 7b) originating MPs. In the case of blood plasma MPs, levels of Cp increased as endotoxemia progressed, peaking at 6 h of inflammation and still remaining high at 24 h (Fig. 7a). In contrary, the content of Cp was rather constant in the case of peritoneal MPs independently of the health status of mice. However, a tendency was observed to increased Cp levels at 24 h of inflammation but it did not reach statistical significance (Fig. 7b).

### Specific inhibition of iNOS activity, but not expression, impacts NO release by MP stimulated macrophages

By application (prior to LPS/MPs stimulation) of non-specific (L-NAME) and specific (1400W) iNOS inhibitors we confirmed that NO production depended on this isoform as NO synthesis ceased upon either of them (Fig. 7c). In these experiments, protein expression of iNOS was unaltered (Fig. 7d, representative images). The same results were obtained when inhibitors were added to the cells after LPS/MP stimulation (data not shown).

## Discussion

Sepsis is a “life-threatening organ dysfunction due to a dysregulated host response to infection” [2] and thus represents a serious/challenging global healthcare problem [46]. In response to systemic inflammation, an overwhelming production of pro-inflammatory and anti-inflammatory cytokines occurs [47] and so does EV release. As EVs are secreted by the majority of cells (leukocytes but also endothelial or epithelial cells), it is not surprising that they are involved in all stages of sepsis, modulating its course. EVs secreted in the early stages of systemic inflammation can protect against vascular hyporeactivity [22], resulting in an improved outcome associated with negative correlation with organ dysfunction and mortality [48]. However, EVs can also act as a double-edged sword, on one hand they protect the endothelium from damage, but on the other hand, they can induce it through scavenging and producing ROS [49] and releasing superoxide anion and nitric oxide [50]. Thus, EVs can also be pro-inflammatory and pro-coagulant [51]. A growing body of evidence suggests that especially one subgroup of EVs—microparticles (MPs), plays a crucial role in inflammation, including initiation and propagation of sepsis. Microparticles derived from platelets, endothelial cells, or leukocytes are upregulated during infection and promote synthesis of mediators of inflammation or act as substrates for their production [52]. In particular, platelet-derived MPs trigger increased recruitment of leukocytes and enhance their aggregation and phagocytic activity [53]. Endothelial MPs induce expression of cell adhesion molecules thus facilitating interaction of leukocytes with endothelium. In turn, MPs derived from polymorphonuclear leukocytes contribute to an increased release of cell adhesion molecules and activation of endothelial cells [53]. Moreover, microparticles isolated from monocytes have been reported to increase cytokine release and superoxide anion production, altogether suggesting that their influence is of key importance for amplification of inflammation [53].

Our current study demonstrates the diverse impact of the MPs originating from different body compartments on murine macrophages. To obtain the microparticles, experimental sepsis was induced in mice (endotoxemia model), and we studied the presence of MPs directly in the vasculature of cremaster muscle with spinning-disk confocal intravital microscopy (IVM) [51]. Cremaster muscle blood vessels are considered to be representative of classical vascular beds [54, 55]. It should be stressed that detection of MPs is possible with IVM via fluorescently labelled antibodies detecting markers of the cells from which MPs originate and they were shown previously in the vasculature of cremaster muscle [56] and the liver [57]. Their subtype known as elongated neutrophil-derived structures and migrasomes, respectively.

As circulating neutrophils and monocytes reach the site of inflammation as the first responders and then initiate successive waves of influx of other immune cells, we focused on observing these two types of leukocytes and MPs released by them, with IVM. Our *in vivo* observations indicate an increased influx of neutrophils and monocytes 8 h post-LPS administration, however, with dominant numbers of neutrophils. In line with this, we see much more neutrophil-derived putative MPs attached to endothelium or rolling along it. However, we observe the opposite pattern when detecting free MPs circulating in blood with NTA. These data indicate that numbers of MPs interacting with endothelium and those freely floating in blood do not correspond with each other, as the majority of MPs of neutrophil origin does adhere to endothelium. Vast majority of MPs is believed to stay in circulating blood, but as they travel [58], they cannot be microscopically identified/spotted due to the high speed of blood components (in perfused vessels the blood sheer is rapid). In our studies, we focused on 8-hour endotoxemia whereas septic patients are tested at much later time points, possible even days after the initiation of systemic inflammation. Although in these patients, neutrophils still predominate over monocytes in blood [59]; often less circulating monocyte-derived MPs [60] are detected. The differences in neutrophil *vs.* monocyte MPs may result from inactivation or dysfunction of monocytes in the course of sepsis as it progresses [60, 61] which is in contrast to overactivation of neutrophils [62]. For example, exposure of peripheral blood mononuclear cells (PBMCs) of trauma patients to LPS decreases expression of the transcription nuclear factor (NF)-κB unlike in those isolated from healthy donors [61]. In fact, during sepsis, monocytes are involved mostly during its early stages whereas later on they downregulate genes involved in inflammation and increase expression of those involved in apoptosis. Thus, the overwhelming cytokine production (cytokine storm) originates rather from resident macrophages than monocytes [23, 63]. This led us to hypothesize that at least some of these phenotypic features of monocytes/macrophages during sepsis/endotoxemia (mimicked by lipopolysaccharide) might be a consequence of MP impact on these cells.

In the course of systemic inflammation, in addition to the primary inflammation (the systemic insult), there may be an additional compartment pathophysiology, and one example being abdominal sepsis [64]. The latter might or might not be connected with peritonitis. For the above reasons, when we subsequently isolated MPs from blood of experimental mice, we have also collected MPs form the peritoneal cavity and analyzed them with NTA for size distribution and overall numbers. To our surprise, peritoneal MP numbers were higher by an order of magnitude from those harvested from blood plasma. Moreover, although we observed heterogeneous MP populations among both blood plasma and peritoneal exudate particles, the pattern was different. The differences in size were more prominent in the case of blood plasma than in peritoneal exudate MPs. On the other hand, MPs isolated from endotoxemic plasma were smaller in size than the peritoneal-derived MPs; however, the average size of dominating population was similar (300.9 *vs*. 318 nm respectively). Furthermore, when we compared their cargo, it turned out that the blood plasma MPs do carry ceruloplasmin in homeostasis but its content increases significantly as sepsis progresses. Ceruloplasmin is one of the major copper-binding glycoproteins present in blood, and its increased levels are observed during pathological states, where it participates in immune response. In particular, Cp carries bactericidal activities, through its ferroxidase activity, it increases iron uptake by cells [65], thus limiting the availability of free iron, which is critical for bacteria to growth and survival [33, 66, 67]. Furthermore, correlation between Cp and other acute phase proteins (C-reactive protein, α-1-antitripsin, α-2-macroglobulin, and alkaline phosphatase) is observed in sepsis [34]. Cp has also been shown to play a protective role by preventing DNA and protein damage in cardiovascular disease [68] as well as from iron-mediated damage in spinal cord contusion injury [69]. Ceruloplasmin oxidizes and thus detoxifies toxic ferrous iron to its nontoxic ferric form. Free iron can generate reactive oxygen and nitrogen species thus leading to noxiously modified nucleic acids and proteins and consequently tissue damage [69, 70]. NO reacts with superoxide radicals (O_2_^−^) to produce peroxynitrite (OONO^−^) that oxidizes proteins, lipids, nitrated amino acids, and DNA [71, 72]. So, it is known that irreversible damage of bioactive molecules is the result of highly reactive secondary species (e.g., peroxynitrite) formed in reaction with a primary species (e.g., nitric oxide) [73]. In line with our studies, Camino et al. [74] reported that ceruloplasmin is up-regulated in extracellular vesicles originating from brown adipose tissue of obese rats as the associated inflammation progresses during obesity development [74]. Moreover, Dalli et al. [75] showed that Cp is observed both in microparticles released during homeostasis and systemic inflammation (severe sepsis/septic shock), and the level of Cp is elevated in neutrophil microparticles obtained from the plasma of septic patients [75]. Interestingly, the multifunctional ceruloplasmin originating from macrophages possesses anti-oxidant properties and contributes to protection against inflammation and tissue damage in acute and chronic experimental colitis [33]. In regard to Cp presence in the peritoneal fluid, the literature is scarce and concentrates on patients post gynecological procedures. Yildirim et al. [76] reported no differences in Cp levels between patients with malignant and benign gynecological pathologies [76]. In turn, Polak et al. [77] showed that in patients with endometriosis (non-infectious peritoneal inflammation) an increase in Cp level was observed only in the subgroup of patients with advanced stages of the disease [77]. The most common comparisons are performed between EVs/MPs originating from the same cell types but released in homeostasis *vs*. pathological conditions. Xu et al. [78] showed that numbers of plasma-derived EVs are much higher in septic patients compared to those collected from healthy volunteers although they are smaller in size [78]. Importantly for meaningful comparisons, the size and number of EVs collected from serum of septic mice (cecal ligation and puncture model, CLP) correspond to human polymicrobial sepsis and so do parameters for healthy counterparts [79].

The main goal of the current study was to verify the impact of MPs on macrophages. Some studies demonstrated that microparticles can change proliferation of RAW 264.7 cells and induce their apoptosis [80]. Moreover, after LPS stimulation, the positive correlation of MPs released and NO production from murine macrophage cell line were observed [81]. The opposite pattern was observed after treatment with an iNOS inhibitor. Interestingly, after treatment of stimulated RAW 264.7 cells with a caspase inhibitor, NO production was lower but MP release increased [81]. Our first analyses ruled out impact of MPs of either blood plasma or peritoneal exudate origin on basic parameters of macrophages such as their adhesion to the surface. In the case of adherent cell lines (as used herein) cell ability to attach to the culture surface is indirectly indicative of their good condition and viability [82]. However, when the cell condition was evaluated by Presto Blue assay that estimates cellular metabolism, MPs of either origin were shown to even increase the cell performance in this respect. Nevertheless, when we verified metabolism with yet another assay, namely, MTT, the cell condition was not significantly altered by the addition of either MPs and independent of their quantity. Although the two tests measure cellular metabolism, they do estimate different intracellular processes [83]. In Presto Blue assay, resazurin reduction occurs only in the cytoplasm of viable cells [84] whereas during MTT assay the substrate (tetrazolium salt) reduction into formazan occurs within mitochondria [84]. For this reason, we conclude that mitochondrial activity is not changed by MPs but their overall metabolic activity might be even improved by the MPs. Foremost, we were interested in the impact of MPs on immunological activities of macrophages, particularly their pro-inflammatory activity in the form of cytokine release, ROS, and reactive nitrogen species (RNS) production. The release of a pro-inflammatory interleukin 1β (IL-1β) was induced by LPS alone, but in cultures supplemented with MPs of either origin and independently of their concentration, its levels dramatically increased (synergistic effect with LPS). IL-1β is one of the major pro-inflammatory cytokines in the course of sepsis/endotoxemia and is produced primarily by monocytes/macrophages [85]. The same effect of MPs was observed in the case of IL-6 for LPS combined with them (independently of their origin and concentration), but the opposite pattern was documented for LPS alone despite the fact that its expression is promoted by IL-1β [86]. However, IL-6 is a pluripotent cytokine with both pro- and anti-inflammatory potentials [87]. In sepsis, overproduction of these two cytokines, among others, contributes to “cytokine storm” leading to sepsis-induced organ dysfunction [85].

Macrophages play a critical role in the pathogen elimination based on their phagocytic activity and execution of the respiratory burst [88]. The latter results in an increased ROS generation which facilitates fight against pathogens; however, it might also be harmful, e.g., as during sepsis due to an imbalance between ROS production and their elimination [88]. Previous studies reported that the release of MPs and overall ROS production positively correlate, not only because ROS contribute to MPs production but also because MPs might be a source of ROS [49]. In our studies, however, we did not detect any changes in ROS production by macrophages in the presence of either concertation of blood plasma or peritoneal MPs. This was not because the cells were unresponsive as they responded to PMA stimulation which leads to an increased respiratory burst [89]. Interestingly, MP cargo might include anti-oxidant molecules involved in ROS scavenging. It was shown that the oxidative stress condition influence both the number and content of MPs released from cells but on the other hand ROS content in the extracellular or intracellular compartments might be modified by MPs [49]. The balance between the two decides if microparticles act in a pro-oxidant (promote ROS production) or an anti-oxidant (ROS scavenging) manner.

The fact that MPs alone do not have an impactful effect on the studied parameters is in line with multiple observations by other groups [90–92]. In general, it is commonly observed that MPs impact cells only in the presence of pathogens, their components (e.g., LPS) or cytokines. It might be that MP cargo interferes with their signaling pathways hence the effect.

Nitric oxide and its derivatives also contribute to macrophage anti-pathogenic arsenal [93]. We detected that microparticles isolated from blood plasma, unlike those from the peritoneal exudate, increased NO synthesis when added to LPS-stimulated macrophages in a concentration-dependent manner. NO synthesis depends on the expression of iNOS [93], and indeed, we detected that blood plasma MPs with LPS increased iNOS expression in macrophages, in line with data on NO release. It was reported previously that MPs originated from endothelial cells upregulated iNOS expression during atherosclerosis which (as sepsis) is characterized by a chronic inflammatory response [94].

Of note, we also report significant differences in NO production dependent on macrophage passage reinforcing caution with which experiments should be performed in this respect. If we were to analyze only data from high passages we would miss the concentration-dependent effect of plasma MPs, in addition to significantly low NO levels released by senescent cells. It is recognized that RAW 264.7 cells differ morphologically, phenotypically and functionally during the consecutive passages and cells from higher passages lose their plasticity and immunological activity [95]. Therefore, this parameter should be strictly controlled for.

In regard to iNOS, surprisingly, we detected its strongest expression induced by a combination of LPS and peritoneal MPs despite the fact that NO production was not enhanced by such a combination of treatments. iNOS expression in leukocytes is regulated by NF-κB [96], but interactions with various proteins can modulate its activity and subsequent NO synthesis [29]. Additionally, iNOS expression can be counteracted preventing its further enzymatic activity; for instance as a consequence of S-nitrosation reaction, irreversible dissociation of holoenzyme dimer occurs due to zinc loss [29]. Consistent with the known oxidative properties of ceruloplasmin through its ferroxidase activity, we believe that Cp present in the microparticles might be an exemplary molecule influencing NO production and iNOS expression. We propose that plasma-originating microparticles which carry significantly more ceruloplasmin upon stimulation with endotoxin can upregulate NO production by RAW 264.7 macrophages. Ceruloplasmin is known to both increase (in microglia; [31]) and decrease NO production (in serum of patients with type II diabetes; [97]), and it can also impact nitric-oxide synthase, again activating it (in microglia; [31]) or inhibiting (in endothelial cells; [98]). Moreover, reduced NO oxidase activity was observed in the blood plasma of ceruloplasmin knockout mice and aceruloplasminemic humans when compared to wild-type animals and healthy volunteers, respectively [99]. In line with our data, Lazzaro et al. [31] showed that ceruloplasmin activated microglia only in combination with LPS and increased production of nitric oxide although without further upregulation of iNOS expression [31]. In our studies, the iNOS expression was further increased—a tendency in the case of blood plasma MPs but statistically significant stronger expression in peritoneal cavity MPs. When we indirectly estimated activity of iNOS (by application of its non-selective and selective inhibitors), it turned out the enzyme expression was unchanged and only NO release was altered in the presence of MPs. But the increase in iNOS expression and activity translated into enhanced NO release only in the case of blood plasma MPs. The differences between plasma and peritoneal MPs might also result from differential packaging of ceruloplasmin in the two types of vesicles or its distinctive oxidative status/activity. Unfortunately, no ceruloplasmin inhibitors exist, and whereas in the case of cells the selective translational silencing can be achieved, no tools are available for inactivation of soluble ceruloplasmin or the one present in MPs.

Taking into account the results obtained by the Zhao et al. [100], which showed that the measured activity of ceruloplasmin oxidase is too low to regulate nitrite production in lung epithelial cells, it was suggested that there is another/unknown enzyme involved in the NO oxidation to nitrite [100]. So, we can speculate that the impaired production of nitric oxide by macrophages in the presence of peritoneum-derived MPs and LPS may result from the lower activity/content of ceruloplasmin (as an exemplary protein impacting oxidation) compared to that present in MPs obtained from plasma. Another possibility is that this protein does not play a role in the production of NO (does not potentiate production of NO) and there is another enzyme which when bound to LPS elicits this response.

## Conclusion

We demonstrated that the application of IVM allows us to visualize MP-like structures during LPS-induced systemic inflammation, and we are able to differentiate between MPs originating from neutrophils and monocytes in the vasculature of the cremaster muscle. Moreover, we show (NTA) that MPs originating from other body compartments than blood differ by numbers, size, and its variations in the population, but most importantly, by their cargo. This in turn indicates that studies should not be limited to blood plasma EVs/MPs due to their high heterogeneity as they all participate *in vivo* in the pathological state(s). Maintaining the balance between secreted MPs with pro- or anti-inflammatory potential may be important in controlling the inflammatory response, any disturbances in the biology of MPs might direct the outcome of sepsis towards either silencing or progression of systemic inflammation. Although we show that MPs do not change macrophage numbers/viability, they impact their mitochondrial activity and pro-inflammatory IL-1β and iNOS expression. The latter might be related to ceruloplasmin loaded in plasma-derived MPs. Interestingly, however, subsequent NO production does not directly correspond to iNOS expression after the addition of MPs of peritoneal fluid origin. Overall, we demonstrate that MPs from different compartments of the body exhibit different biological properties, which effects are observed in cell communication/response both in homeostasis and in pathological states.

### Supplementary information

ESM 1 (PDF 84 kb)

## Data Availability

The datasets used and/or analyzed during the current study are available from the corresponding author on reasonable request.
