# Supplementary material for: Impact of microparticles released during murine systemic inflammation on macrophage activity and reactive nitrogen species regulation
Source: Immunol Res. 2023 Nov 27;72(2):299–319. doi: 10.1007/s12026-023-09436-7 (PMC11031483; doi:10.1007/s12026-023-09436-7)
Supplement: Supplementary file 1 — (PDF 84 kb) [file 12026_2023_9436_MOESM1_ESM.pdf]

Supplementary Figure S1

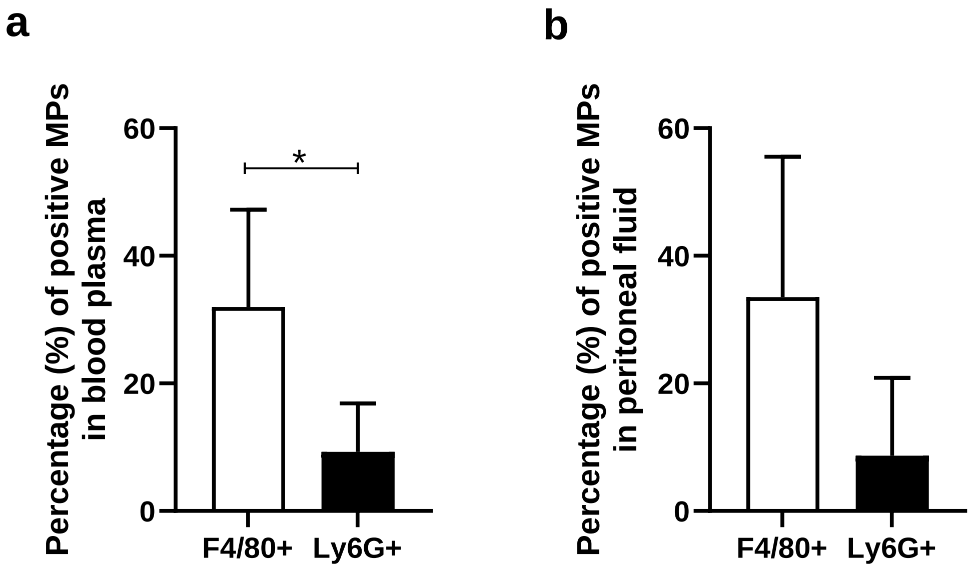

Supplementary Fig. S1 Secretion of microparticles (MPs) positive for F4/80^{+} (monocyte/macrophage origin) and Ly6G^{+} (neutrophil origin) in body fluids as detected by flow cytometry**.** MPs were isolated from (a) blood (plasma) and (b) peritoneal fluid of C57BL/6J mice with LPS-induced endotoxemia (1 mg/kg b.w.; 8 hr post LPS inoculation). The results are expressed as the mean values ± SD. Data were analyzed with unpaired two-tailed Student’s *t*-test (* $p \leq 0.05$).
